# Pirtobrutinib, a highly selective, noncovalent (reversible) BTKi in R/R marginal zone lymphoma: phase 1/2 BRUIN study

**DOI:** 10.1182/bloodadvances.2025017489

**Published:** 2026-01-20

**Authors:** Krish Patel, Julie M. Vose, Sunita D. Nasta, Jennifer R. Brown, Kami J. Maddocks, Jennifer A. Woyach, Nirav N. Shah, Bita Fakhri, Benoit Tessoulin, Shuo Ma, Deepa Jagadeesh, Ewa Lech-Maranda, Catherine C. Coombs, Manish R. Patel, Joanna M. Rhodes, Chaitra Ujjani, Marc S. Hoffmann, Chan Y. Cheah, Talha Munir, David Lewis, Lydia Scarfò, Toby A. Eyre, Alvaro Alencar, Jonathon B. Cohen, Andrew D. Zelenetz, Donald E. Tsai, Mei Li, Yuanyuan Bian, Paolo Abada, Minna Balbas, Pier Luigi Zinzani

**Affiliations:** 1 Swedish Cancer Institute, Seattle, WA; 2 Division of Hematology, University of Nebraska Medical Center, Omaha, NE; 3 Abramson Cancer Center, University of Pennsylvania, Philadelphia, PA; 4 Dana-Farber Cancer Institute and Harvard Medical School, Boston, MA; 5 The Ohio State University Comprehensive Cancer Center, Columbus, OH; 6 Division of Hematology/Oncology, Medical College of Wisconsin, Milwaukee, WI; 7 Division of Hematology, Stanford University School of Medicine, Stanford, CA; 8 Haematology Department, University Hospital of Nantes, Nantes, France; 9 Robert H. Lurie Comprehensive Cancer Center of Northwestern University, Chicago, IL; 10 Department of Hematology and Medical Oncology, Cleveland Clinic, Cleveland, OH; 11 Institute of Hematology and Transfusion Medicine, Warsaw, Poland; 12 Division of Hematology/Oncology, University of California Irvine, Irvine, CA; 13 Florida Cancer Specialists, Sarasota, FL; 14 Sarah Cannon Research Institute, Nashville, TN; 15 Rutgers Cancer Institute of New Jersey, New Brunswick, NJ; 16 Division of Hematology and Oncology, Fred Hutchinson Cancer Research Center, Seattle, WA; 17 The University of Kansas Cancer Center, Kansas City, MO; 18 Linear Clinical Research and Sir Charles Gairdner Hospital, Nedlands, WA, Australia; 19 Department of Haematology, St James's University Hospital, Leeds, United Kingdom; 20 University Hospitals Plymouth NHS, Plymouth, United Kingdom; 21 Università Vita-Salute San Raffaele and IRCCS Ospedale San Raffaele, Milan, Italy; 22 Oxford University Hospitals NHS Foundation Trust, Churchill Cancer Center, Oxford, United Kingdom; 23 University of Miami Sylvester Comprehensive Cancer Center, Miami, FL; 24 Winship Cancer Institute, Emory University, Atlanta, GA; 25 Department of Medicine, Lymphoma Service, Memorial Sloan Kettering Cancer Center, New York, NY; 26 Eli Lilly and Company, Indianapolis, IN; 27 Istituto di Ematologia “Seràgnoli,” Istituto di Ricovero e Cura a Carattere Scientifico Azienda Ospedaliero-Universitaria di Bologna, Bologna, Italy; 28 Dipartimento di Scienze Mediche e Chirurgiche, Università di Bologna, Bologna, Italy

## Abstract

Marginal zone lymphoma (MZL) is a group of indolent B-cell malignancies with a remitting and relapsing course. For systemic disease, available first-line therapies include anti-CD20 antibody as monotherapy or in combination with chemotherapy (chemoimmunotherapy), with second-line options including covalent Bruton tyrosine kinase inhibitors (cBTKi). However, management of relapsed and refractory (R/R) MZL remains challenging. Pirtobrutinib, a highly selective, noncovalent BTKi has shown promising efficacy and tolerability in poor-prognosis B-cell malignancies following prior therapy, including cBTKi. Here, we report the safety and efficacy of pirtobrutinib in MZL from the phase 1/2 BRUIN study. Endpoints included investigator-assessed objective response rate (ORR) by Lugano 2014 criteria, duration of response (DOR), progression-free survival (PFS), overall survival (OS), and safety. Among 36 patients with R/R MZL (extranodal [n = 6]; nodal [n = 17]; splenic [n = 13]), median age was 68 years (range, 22-83) and median prior lines of therapy were 3 (range, 2-10). The ORR was 55.6% (95% confidence interval [CI], 38.1- 72.1), including 3 (8.3%) complete responses and 17 (47.2%) partial responses. Median DOR was 17.8 months (95% CI, 7.4 to nonestimable [NE]), and median PFS was 16.6 months (95% CI, 9.0-22.1). With median follow-up of 32.4 months (IQR, 28.0-41.3), median OS was NE (95% CI, 29.5-NE). The ORR for patients with prior cBTKi therapy was 53.8% (95% CI, 33.4-73.4). Pirtobrutinib was well tolerated with dose reductions in 4 patients (11.1%) and permanent discontinuation due to treatment emergent adverse events in 4 patients (11.1%). Pirtobrutinib showed promising efficacy and safety in patients with heavily pretreated R/R MZL, including prior cBTKi. This trial was registered at www.clinicaltrials.gov as #NCT03740529.

## Key Points

• Pirtobrutinib appears to offer clinical benefit in R/R MZL, with ORR of 55.6% and median DOR of 17.8 months.
• Pirtobrutinib shows a favorable safety profile in R/R MZL, with low rates of dose reduction (11.1%) and discontinuation (11.1%) due to AEs.

## Visual Abstract

**Figure:**
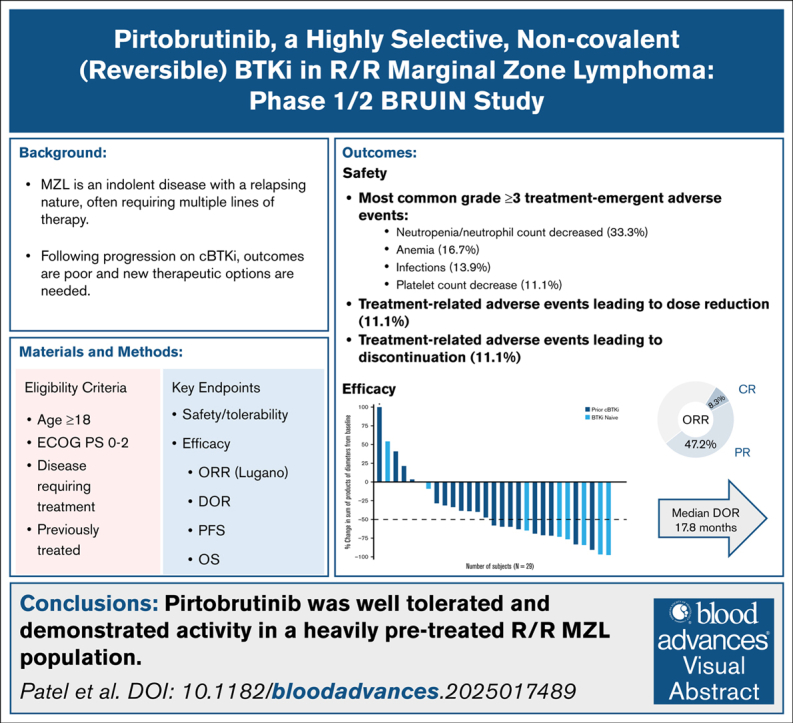

## Introduction

Marginal zone lymphoma (MZL) is an indolent non-Hodgkin lymphoma (NHL) accounting for ∼7% of all NHL cases.^1^ It is thought to originate from B cells of the postgerminal center marginal zone that may be associated with an underlying infectious etiology, subsequent induction of B-cell receptor signaling and consequently abnormal cell proliferation and survival.^2^^,^^3^ Key subtypes of MZL include extranodal MZL (EMZL), such as mucosa-associated lymphoid tissue lymphoma), that accounts for approximately two-thirds of all cases, splenic MZL (SMZL) which accounts for ∼20% and nodal MZL (NMZL), which accounts for <10% of all cases.^4^

Although prognosis can vary between subtypes, favorable outcomes can be achieved for most patients with systemic treatment.^5^ First-line systemic treatment options across MZL subtypes typically include rituximab monotherapy or rituximab in combination with chemotherapy.^6^, ^7^, ^8^ An overall response rate (ORR) of 82% has been reported among patients with previously untreated MZL utilizing the immunochemotherapy approach.^9^ Despite most patients achieving long durations of disease control with initial treatments, the majority will relapse, and there are limited treatment regimens for subsequent lines of treatment, and options become reduced.^5^^,^^10^^,^^11^

In recent years, novel agents like covalent Bruton tyrosine kinase inhibitors (cBTKi) have a growing role in the management of B-cell malignancies, especially in chronic lymphocytic leukemia (CLL) and mantle cell lymphoma.^12^^,^^13^ In the R/R MZL setting immunomodulators such as lenalidomide and cBTKi such as zanubrutinib, acalabrutinib, and ibrutinib, have increasingly been used with success.^5^ Results from the AUGMENT study showed that a combination of lenalidomide and rituximab had promising efficacy in patients with R/R MZL (ORR, 65%; complete response [CR], 29% and median progression-free survival [PFS] as assessed by IRC of 20.2 months).^14^ In a BTKi-naïve population, monotherapy with zanubrutinib resulted in IRC-assessed ORR of 68.2% (25.8% CR),^15^ whereas treatment with acalabrutinib and ibrutinib resulted in ORR of 52.5% (12.5% CR)^16^ and 48% (3% CR),^17^ respectively. cBTKi have not been curative, and acquired mutations that confer resistance to BTKi or intolerance to these agents pose a challenge for the subsequent treatment with other BTKi agents in patients with R/R MZL.^18^^,^^19^ Limited data exist in the post-cBTKi setting with trials excluding patients previously exposed to cBTKi^15^ but existing data suggest poor outcomes.^20^ In a phase 2 trial of axicabtagene ciloleucel, a chimeric antigen receptor (CAR) T-cell therapy, in patients with R/R indolent NHL, with a median follow-up of 64.6 months, the median duration of response (DOR) was not reached and the 60-month DOR rate was 60.0% in the MZL cohort (n = 31).^21^ Although CAR T-cell therapy can demonstrate durable responses in a MZL population, the treatment can be challenging to administer, and data is lacking in the post-BTKi setting and has not received regulatory approval. Due to the lack of studies in post-cBTKi patients with R/R MZL and the need for treatment options for patients that relapse, there remains a clear need to investigate other therapies for these patients with the aim of improving patient outcomes in the R/R setting.

Pirtobrutinib, a highly selective, noncovalent (reversible) BTKi, that inhibits both wildtype and C481-mutant BTK with equal low nanomolar potency, has favorable oral pharmacology that enables continuous BTK inhibition throughout the once-daily dosing interval.^22^ In results from the phase 1/2 BRUIN study, pirtobrutinib demonstrated promising efficacy and tolerability in heavily pretreated patients with poor-prognosis B-cell malignancies, including those treated with a prior cBTKi.^23^^,^^24^ These data led to the approvals of pirtobrutinib in the United States and Europe EU in chronic lymphocytic leukemia/small lymphocytic lymphoma (CLL/SLL) and mantle cell lymphoma.^25^, ^26^, ^27^ Here we report the safety and efficacy of pirtobrutinib in 36 patients with MZL from the BRUIN phase 1/2 study.

## Methods

### Study design

Patients assigned by study phase and B-cell malignancy are shown in supplemental Figure 1. Doses ranging from 25 to 300 mg once daily were explored in phase 1 of this trial with patients in the R/R MZL cohort treated in either dose escalation or dose expansion portions. Two patients in the phase 1 portion of the study received 100 mg and 250 mg daily. The recommended phase 2 dose (RP2D) of 200 mg once daily used in the phase 2 part of this trial was received by 34 patients. Treatment was administered until disease progression, discontinuation because of toxicity, or patient/physician decision to withdraw. Patients with disease progression were permitted to continue pirtobrutinib treatment at the investigator’s discretion if clinical benefit was experienced. The trial protocol was approved by the institutional review boards overseeing each participating site. The trial was conducted in accordance with the Declaration of Helsinki, Good Clinical Practice guidelines, and local laws. All patients provided written informed consent. This trial was registered with ClinicalTrials.gov as #NCT03740529.

### Patients

The eligibility criteria for the complete patient cohort enrolled in the phase 1/2 BRUIN trial have previously been described.^22^ Patients in the MZL cohort were ≥18 years of age, with an Eastern Cooperative Oncology Group Performance Status score of 0 to 2, had R/R disease and had histologically confirmed active MZL. There was no limit on prior lines of therapy. Patients who had received prior cBTKi-containing regimens were eligible for this study.

Pirtobrutinib was administered to patients in oral tablet form at the assigned dose. Dosing was at a consistent time each day. Cycle length was 28 days. Disease evaluations were carried out every 8 weeks in the first year, every 12 weeks in the second year and every 6 months thereafter. Peripheral blood and bone marrow biopsies were collected and used to assess response.

### Outcomes

Efficacy end points for patients with MZL as evaluated by investigators were ORR, DOR, and PFS. Overall survival (OS) was also assessed. ORR was determined according to the Lugano 2014 Treatment Response Criteria including evaluation of CR, partial response , stable disease (SD) and progressive disease (PD).^28^ DOR was calculated for patients who achieved a response of PR or better. For such patients, DOR is defined from the start date of first documented response to the earlier of the documentation of definitive PD or death from any cause. PFS was measured from the treatment start date until the first date of progression or death from any cause. Patients without progression or death were censored at the last adequate disease assessment, and those who started a subsequent anticancer therapy prior to progression or death were censored at the last adequate disease assessment before the start of subsequent anticancer therapy. OS was measured from treatment start date until death from any cause. Patients who were alive and lost to follow-up were censored at the date last known alive.

Treatment emergent adverse events (TEAE) were defined as any adverse event (AE) reported from the date of the first dose through the last dose date + 37 days or start of subsequent anticancer therapy, whichever was earlier. Frequency and severity of TEAEs were evaluated and graded according to the NCI Common Terminology Criteria for Adverse Events (CTCAE, version 5.0). The reported AE term is coded using version 26.0 of the Medical Dictionary for Regulatory Activities.

### Statistical analyses

The data cut-off was 27 January 2025. Descriptive statistics were used to summarize patient baseline demographics, disease characteristics, best overall response (BOR), and safety data. ORR was estimated with an exact, 2-sided 95% confidence interval (CI). The Kaplan-Meier method was used to estimate distributions of DOR, PFS, and OS. All analyses were conducted using SAS version 9.4.

## Results

### Patients

A total of 36 patients were enrolled to the MZL cohort. Baseline patient and disease characteristics are described in Table 1. Patients had a median age of 68 years (range, 22-83). Over half of the patients were female (56%, n = 20), and median prior lines of systemic therapy were 3 (range, 2-10). Most of the patients had NMZL subtype (47%, n = 17), followed by SMZL (36%, n = 13), and EMZL (17%, n = 6). More than half of the patients at the time of study enrollment were in the high-risk MALT-IPI (Mucosa-Associated Lymphoid Tissue–International Prognostic Index) group (53%, n = 19), 10 (28%) were intermediate, 1 (3%) was low, and missing in 6 (17%) patients. Baseline lactate dehydrogenase was elevated in 15 (42%) patients and hemoglobin was <12 g/dL in 29 (81%) patients. All 36 patients with MZL were previously treated with an anti-CD20 antibody, and 31 (86%) patients had received prior chemotherapy. Of the 26 patients (72%) with MZL who received a prior cBTKi, 20 (77%) discontinued due to PD and 6 (23%) discontinued due to toxicity/other reasons.

**Table 1.** **Patient baseline characteristics**

Characteristics	N = 36
Median age (range), y	68 (22-83)
Male, n (%)	16 (44.4)
Female, n (%)	20 (55.6)
**ECOG PS**, **n (%)**	
0	18 (50)
1	17 (47.2)
2	1 (2.8)
**MZL subtype**, **n (%)**	
Nodal	17 (47.2)
Splenic	13 (36.1)
Extranodal∗	6 (16.7)
**Tumor bulk, n (%)**	
≥5 cm	5 (13.9)
<5 cm	23 (63.9)
No measurable lymph node	8 (22.2)
**Elevated LDH, n (%)**	
Yes	15 (41.7)
No	21 (58.3)
**Baseline hemoglobin <12 g/dL**	
Yes	29 (80.6)
No	7 (19.4)
**Involved nodal sites, n (%)**	
≤4	20 (55.6)
>4	16 (44.4)
**Ann Arbor staging, n (%)**	
Stage I/II	1 (2.8)
Stage III/IV	29 (80.6)
Missing	6 (16.7)
**MALT-IPI risk group**, **n (%)**	
Low risk (0)	1 (2.8)
Intermediate risk (1)	10 (27.8)
High risk (≥2)	19 (52.8)
Missing	6 (16.7)
Median number of prior lines of systemic therapy (range)	3 (2-10)
**Prior therapy, n (%)**	
BTK inhibitor	26 (72.2)
Ibrutinib	17 (47.2)
Acalabrutinib	1 (2.8)
Zanubrutinib	8 (22.2)
Other	0
Anti-CD20 antibody	36 (100)
Chemotherapy and Anti-CD20 antibody	31 (86.1)
PI3K inhibitor	6 (16.7)
Lenalidomide	8 (22.2)
BCL2 inhibitor	1 (2.8)
Autologous stem cell transplant	1 (2.8)
Other systemic therapy†	4 (11.1)
**Reason for discontinuation of any prior cBTKi**‡**,**§, **n (%)**	
Progressive disease	20 (55.6)
Toxicity/other	6 (16.7)

Data cutoff of 27 January 2025.

BCL2, B-cell lymphoma 2; CAR-T, chimeric antigen receptor T-cell therapy; ECOG PS, Eastern Cooperative Oncology Group performance status; LDH, lactate dehydrogenase; PI3K, phosphoinositide 3-kinase; ULN, upper limit of normal.

∗ Extranodal MZL of mucosa-associated lymphoid tissue.

† Other systemic therapies include: histone deacetylase inhibitors, radioimmunotherapy, anti-CD74 antibodies, and proteasome inhibitors.

‡ Percentage related to the population of patients previously treated with another cBTKi.

§ In the event more than one reason was noted for discontinuation, disease progression took priority.

### Efficacy

**Figure 1.**
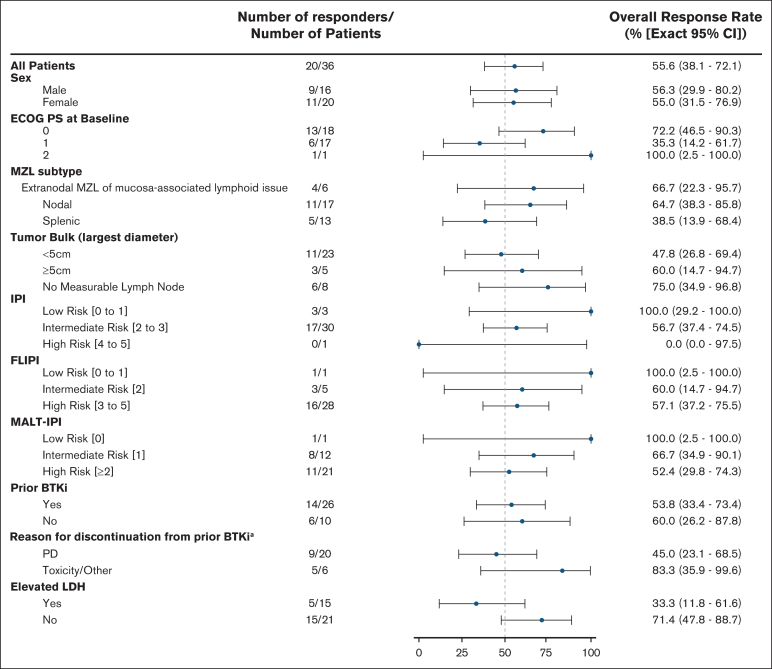
**ORR in Patient Subgroups Investigator-assessed ORR per Lugano 2014 criteria by subgroups.** Data cutoff of 27 January 2025. ^a^In the event more than 1 reason was noted for discontinuation, disease progression took priority. BCL2, B-cell lymphoma 2; CAR-T, chimeric antigen receptor T-cell therapy; ECOG PS, Eastern Cooperative Oncology Group performance status; IPI, international prognostic index; LDH, lactate dehydrogenase; MALT, mucosa-associated lymphoid tissue; PI3K, phosphoinositide 3-kinase; ULN, upper limit of normal.

The ORR among all patients with MZL was 55.6% (95% CI, 38.1- 72.1) (Figure 1), including 3 (8.3%) CR and 17 (47.2%) PR (Table 2). The ORR in patients with EMZL, NMZL, and SMZL were 66.7% (n = 4/6, 95% CI, 22.3-95.7), 64.7% (n = 11/17; 95% CI, 38.3-85.8), and 38.5% (n = 5/13; 95% CI, 13.9-68.4), respectively. The ORR for patients with MZL with prior cBTKi therapy was 53.8% (n = 14/26; 95% CI, 33.4-73.4). The ORR for patients who had discontinued prior cBTKi treatment due to disease progression and toxicity/other reasons were 45.0% (n = 9/20; 95% CI, 23.1-68.5) and 83.3% (n = 5/6; 95% CI, 35.9-99.6), respectively (Figure 1).

**Table 2.** **Best overall response among patients with R/R MZL treated with BTKi therapies**

	All MZL patients N = 36	Prior cBTKi patients n = 26	cBTKi naïve n = 10
ORR∗, % (95% CI)	55.6 (38.1, 72.1)	53.8 (33.4, 73.4)	60 (26.2, 87.8)
**Best response**, n (%)			
CR	3 (8.3)	1 (3.8)	2 (20)
PR	17 (47.2)	13 (50.0)	4 (40)
SD	13 (36.1)	10 (38.5)	3 (30)
PD	3 (8.3)	2 (7.7)	1 (10)

Data cutoff of 27 January 2025.

∗ ORR is the number of patients with best response of CR or PR divided by the total number of patients.

**Figure 2.**
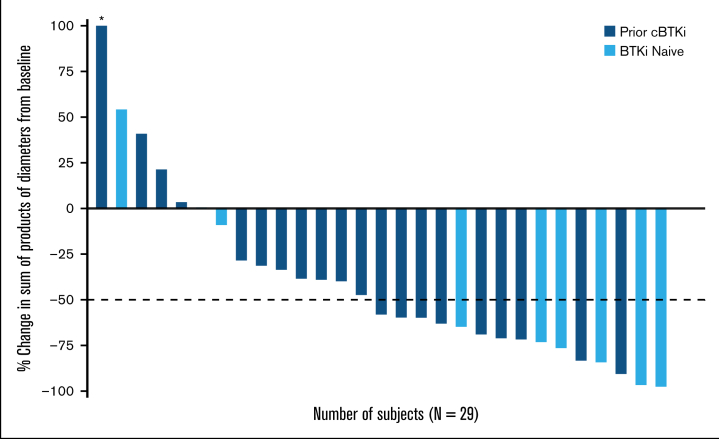
**Pirtobrutinib efficacy in patients with MZL.** Best change in sum of product of diameters from baseline (A) and time to first response among patients with response (B) in MZL patients. Data for 7 patients are not shown in the waterfall plot (A) due to no measurable target lesions identified by CT scan at baseline. Data cutoff of 27 January 2025. ∗Patient with a >100% increase in sum of products of diameter, with the corresponding change from baseline of 181.6%.

As shown in the waterfall plot (Figure 2) among patients with baseline and postbaseline tumor assessment (n = 29), 79.3% (n = 23) of patients achieved a reduction in sum of product of tumor diameters from baseline, with similar reduction observed in cBTKi-naïve patients (77.8%; [n = 7/9]) and patients previously treated with cBTKi (80%; [n = 16/20]) (Figure 2).

**Figure 3.**
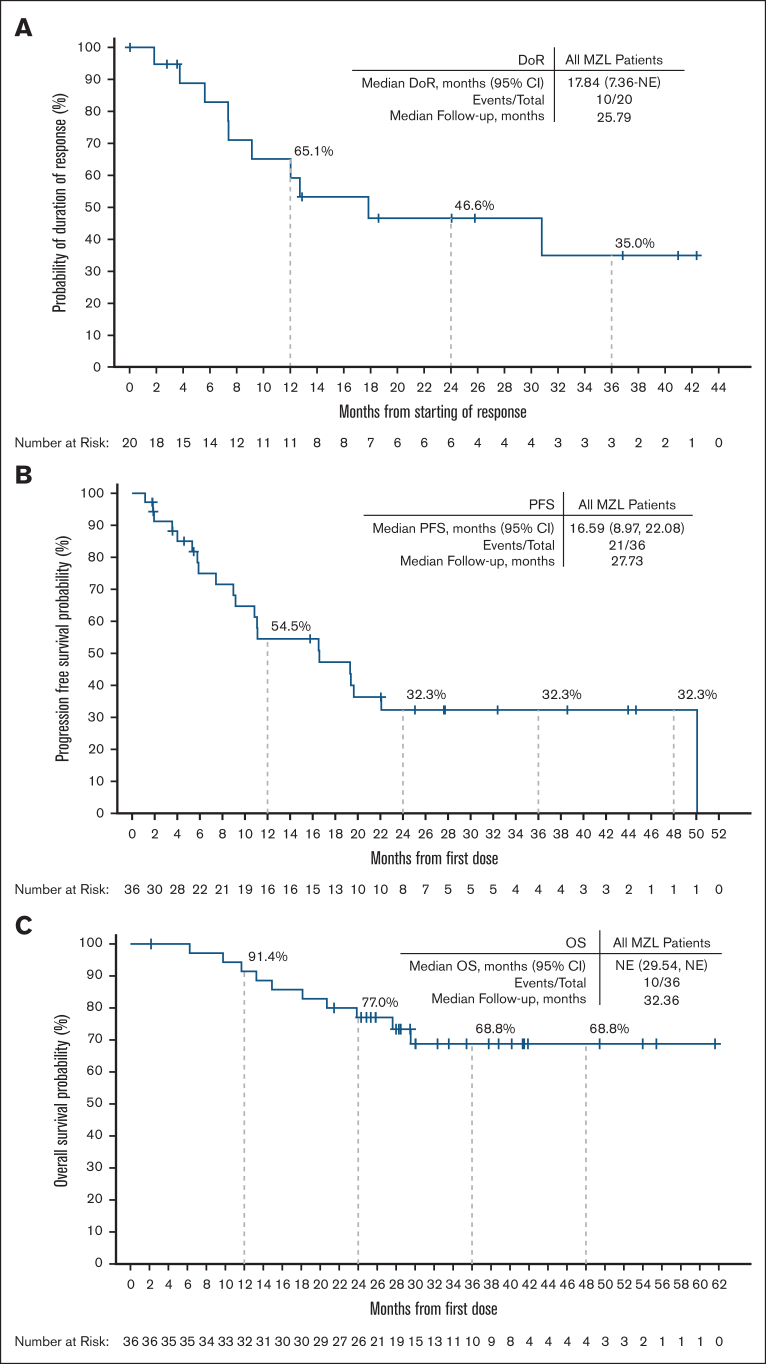
**DOR, PFS, and OS among patients with R/R MZL.** Kaplan-Meier curves representing investigator-assessed DOR (A) and PFS (B) per Lugano 2014 criteria, and (C) OS of all patients with R/R MZL enrolled in the study. Patients who were alive and without documented PD (DOR, PFS) as of the data analysis cutoff date were censored. Patients who were alive or lost to follow-up (OS) as of the data analysis cutoff date were censored. Data cutoff of 27 January 2025.

**Figure 4.**
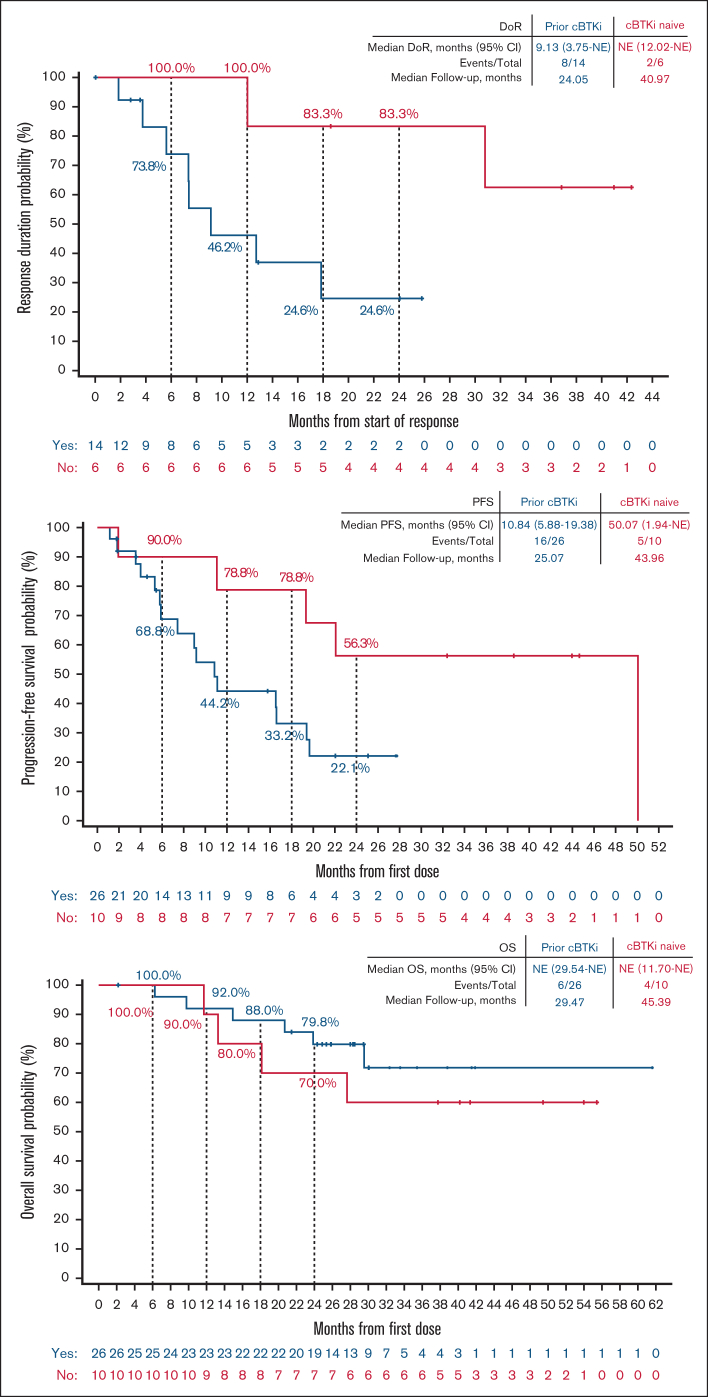
**DOR, PFS and OS among patients with R/R MZL by prior cBTKi treatment status.** Kaplan-Meier curves representing Investigator-assessed DOR (a) and PFS (b) per Lugano 2014 criteria, and OS (c) in patients with R/R MZL based on prior cBTKi treatment status. Data cutoff of 27 Jan 2025. Abbreviations: cBTKi, covalent Bruton tyrosine kinase inhibitor; CI, confidence interval; DoR, duration of response; MZL, marginal zone lymphoma; NE, not estimable; OS, overall survival; PFS, progression free survival.

Among responding patients, the median time to response was 1.9 months (range, 1.6-19.3), corresponding to the timing of the initial response assessment (supplemental Figure 3). The median DOR was 17.8 months (95% CI, 7.4 to nonestimable [NE]) with a median follow-up time of 25.8 months. The 24-month DOR of 46.6% (95% CI, 22.3-67.9) (Figure 3A). Among patients previously treated with cBTKi (n = 14), the median DOR was 9.1 months (95% CI, 3.8-NE) (Figure 4A).

The median PFS for the whole cohort was 16.6 months (95% CI, 9.0-22.1) with a median follow-up of 27.7 months, and PFS rate of 32.3% at 24 months (95% CI, 16.3-49.5) (Figure 3B). The median PFS of EMZL, NMZL and SMZL subtypes, respectively, was NE (95% CI, 7.43-NE), 11.1 months (95% CI, 4.0-NE), and 16.6 months (95% CI, 1.9-22.1) (supplemental Figure 2). The median PFS in patients with prior cBTKi treatment was 10.8 months (95% CI, 5.9-19.4) (Figure 4B). For patients who had discontinued prior cBTKi treatment due to disease progression (20 patients) and toxicity/other reasons (6 patients), the median PFS was 9.17 months (95% CI, 5.32-16.59) and 19.38 (95% CI, 8.97, NE), respectively (supplemental Figure 4).

The median OS for the whole cohort was NE (95% CI, 29.5-NE) with a median follow-up of 32.4 months. The 24-month OS rate was 77.0% (95% CI, 59.3-87.8; Figure 3C). In patients previously treated with a cBTKi, the median OS was also NE (95% CI, 29.5-NE) (Figure 4C).

### Safety

Median time on treatment was 15.4 months (range, 1.1-61.6), with 8 (22.2%) patients still receiving pirtobrutinib at the time of the data cut-off. Fifteen (41.7%) patients discontinued the study, and 6 (16.7%) patients completed the study follow-up at the time of the data cut-off.

TEAEs were experienced by 35 patients (97.2%). The most frequent TEAEs, treatment-related AE (TRAE) and AEs of interest are shown in Table 3. The most common any grade TEAEs regardless of attribution were diarrhea (44.4%, n = 16), fatigue (33.3%, n = 12), neutropenia/neutrophil count decreased (36.1%, n = 13), anemia (33.3%, n = 12), and dyspnea (30.6%, n = 11). Grade ≥3 TEAEs were experienced by 23 (63.9%) patients with MZL. The most frequent grade ≥3 TEAEs were neutropenia/neutrophil count decreased (33.3%, n = 12), anemia (16.7%, n = 6), infections (13.9%, n = 5), and platelet count decrease (11.1%, n = 4).

**Table 3.** **Pirtobrutinib safety profile in MZL patients**

	Treatment emergent AEs in patients with MZL (N = 36)			
	All-cause AEs, (≥20%) %		Treatment-related AEs, %	
AE	Any grade	Grade ≥3	Any grade	Grade ≥3
Diarrhea	44.4	5.6	19.4	2.8
Fatigue	33.3	0	13.9	0
Neutropenia∗	36.1	33.3	13.9	13.9
Anemia	33.3	16.7	8.3	5.6
Dyspnea	30.6	0	2.8	0
Nausea	25.0	0	2.8	0
Abdominal Pain	25.0	0	0	0
Cough	25.0	0	8.3	0
COVID-19	22.2	2.8	2.8	0
Platelet count decrease	22.2	11.1	11.1	2.8
AEs of interest†	Any grade	Grade ≥3	Any grade	Grade ≥3
Infection‡	63.9	13.9	13.9	0
Bruising§	30.6	0	25.0	0
Rash||	27.8	0	19.4	0
Hemorrhage¶	13.9	0	0	0
Hypertension	8.3	2.8	2.8	2.8
Atrial Fibrillation/flutter#	0	0	0	0

Data cutoff of 27 January 2025.

∗ Aggregate of neutropenia and neutrophil count decreased.

† AEs of interest are those previously associated with cBTKi.

‡ Aggregate of all preferred terms indicating infection and including COVID-19.

§ Includes preferred term of contusion.

|| Aggregate of all preferred terms indicating rash.

¶ Aggregate of all preferred terms including hemorrhage and hematoma.

# Aggregate of atrial fibrillation and atrial flutter.

A total of 4 (11.1%) patients had an AE that required dose reduction, and 4 (11.1%) patients discontinued treatment due to an AE. Two (5.6%) patients discontinued pirtobrutinib due to a treatment-related AE (diarrhea in 1 patient, and neutropenia and platelet count decreased in the other), and neither had received a prior BTKi. The 2 patients who discontinued due to an unrelated AE had received a prior BTKi and both had discontinued prior BTKi due to disease progression. No fatal TEAEs were observed.

AEs commonly associated with cBTKi therapy such as hemorrhage/hematoma, arthralgia, rash, and bruising of any grade occurred in 13.9% (n = 5); 19.4% (n = 7); 27.8% (n = 10) and 30.6% (n = 11) of patients, respectively. Grade ≥3 hemorrhage/hematoma, arthralgia, rash, and bruising were not observed. Any grade hypertension (8.3%, n = 3) was infrequent. No atrial fibrillation/flutter was observed.

## Discussion

Available treatments can help maintain long-term disease control in patients with MZL; however, most will likely relapse, leaving limited options for subsequent treatments.^5^^,^^10^^,^^11^ Immunomodulators like lenalidomide have demonstrated promise and have been included in treatment guidelines.^29^ The AUGMENT study showed promising efficacy of combining lenalidomide and rituximab, which resulted in an ORR of 65% in patients with R/R MZL.^14^ Zanubrutinib, ibrutinib, and acalabrutinib were extensively studied in BTKi-naïve MZL settings (both R/R and treatment naïve), and demonstrated activity (ORR range, 48%-74.2%).^15^, ^16^, ^17^^,^^19^^,^^30^ For example, acalabrutinib in the treatment of R/R MZL resulted in ORR of 52.5% [95% CI, 36.1-68.5) and PFS of 27.4 months (95% CI, 11.1 months to NE),^16^ in a BTKi-naive population. As a result, lenalidomide and BTKi-based treatments have been included in the treatment guidelines as second-line and subsequent therapies.^31^

However, outcomes after cBTKi treatment have been poor,^32^ and highly efficacious and well-tolerated treatment options for relapsed patients in this specific setting are lacking. CAR T-cell therapy, which is included in the treatment guidelines as an option after cBTKi therapy,^31^ has demonstrated durable responses with a 60-month DOR rate of 60.0% in patients in R/R MZL.^21^ However, CAR T-cell therapies are not well suited for many patients and have not yet been approved by the regulatory authorities; thus, they are not yet widely available, and data in a post-BTKi setting are lacking.

Pirtobrutinib showed promising efficacy as monotherapy in this cohort of heavily pretreated patients with R/R MZL. An ORR of 55.6% was observed among all patients treated with pirtobrutinib, with similar efficacy in patients who received prior cBTKi therapy (ORR, 53.8%). Although lower ORR was observed in the SMZL subgroup, the small number of patients limit the ability to draw clear conclusions about efficacy in each subtype. Benefits appeared durable, with patients having a median DOR of 17.8 months and a median PFS of 16.6 months. In addition, 5 patients with a BOR of SD experienced extended PFS (25.07, 22.08, 19.38, 11.10, and 11.07 months). One additional patient with a BOR of SD had PFS of 9.17 months but continued on treatment for 32 months after progression. Pirtobrutinib was also well tolerated with minimal reports of AEs frequently associated with cBTKi as well as low rates of discontinuations due to AEs (11.1%, n = 4). Overall, the safety profile of pirtobrutinib was favorable and comparable to the larger population of patients with different B-cell malignancies treated with pirtobrutinib in the BRUIN phase 1/2 study.^22^

Molecular associations in studies using cBTKi have been identified in MZL which can affect treatment response and resistance. Exploratory analysis in the phase 2 PCYC-1121 study identified mutations in genes such as *MYD88*, *TNFAIP3*, and *KMT2D* to be associated with PFS in R/R MZL.^19^ In particular, patients with *MYD88* or *TNFAIP3* mutations presented a better response to ibrutinib, whereas *KMT2D* and *CARD11* mutations were associated with worse clinical outcomes.^19^ Moreover, mutations in both *BTK* (C481S) and *PLCG2* have been identified as conferring resistance to cBTKi in patients receiving ibrutinib or zanubrutinib.^18^^,^^33^ In this MZL cohort, *BTK* C481x and *PLCG2* mutations were not evaluated. However, investigation of the efficacy of pirtobrutinib in the setting of acquired resistance to cBTKi has been performed in other B-cell malignancies and may provide further insight into the use of pirtobrutinib in the MZL population.

Pirtobrutinib has shown favorable pharmacokinetics, with high oral bioavailability and an ∼20-hour half-life, achieving continuous BTK inhibition throughout the dosing interval, regardless of the intrinsic rate of BTK turnover. The highly selective nature of pirtobrutinib can also reduce off-target inhibition, consequently minimizing AEs while allowing maximal on-target drug coverage.^22^ Previous studies have demonstrated that pirtobrutinib may stabilize BTK in a closed, inactive conformation leading to fewer interactions with cellular proteins than cBTKi-bound BTK, limiting kinase-independent BTK cellular signaling. These unique characteristics may contribute to the broad efficacy and safety of pirtobrutinib in R/R MZL even after treatment with cBTKi.

Pirtobrutinib has shown activity in patients with MZL who have failed or is intolerant to cBTKi and may provide a therapeutic option for this patient population. The results presented here highlight pirtobrutinib as a valuable treatment option in R/R MZL, and currently pirtobrutinib is included in the NCCN guidelines as a preferred therapy for the treatment of MZL after prior cBTKi.^31^ These data hint at clinical benefit of pirtobrutinib in MZL; however, further evaluation with larger sample sizes is warranted to study pirtobrutinib both as monotherapy and in combination. In fact, there is an ongoing phase 2 study evaluating the efficacy of pirtobrutinib in combination with rituximab in patients with untreated MZL (NCT06390956; PIONEER-MZL). This study may contribute to elucidating the potential benefit of pirtobrutinib as a combination partner in earlier therapy lines.

This trial has some important limitations including the small number of patients with R/R MZL enrolled in the study (n = 36) which precluded robust analysis of subgroups and lack of correlative BTK mutational or other genomic analyses. Moreover, the BRUIN phase 1/2 clinical trial was an open-label, single-arm study that lacked an active control group; therefore, direct comparison to other available therapies is not possible.

### Conclusions

In summary, pirtobrutinib, a first-in-class, noncovalent (reversible) BTKi, showed promising efficacy and was well tolerated with low rates of discontinuation in heavily pretreated patients with R/R MZL, including those previously treated with other cBTKi agents. Pirtobrutinib monotherapy is a promising chemotherapy-free therapeutic option after cBTKi treatment in patients with R/R MZL.

Conflict-of-interest disclosure: K.P. reports consulting or advisory role for AstraZeneca, Genentech, BeiGene, Pharmacyclics, BMS/Celgene/Juno, MorphoSys, Kite, a Gilead company, TG Therapeutics, Loxo/Lilly, AbbVie, Seagen, Epizyme, ADC Therapeutics, Caribou Biosciences, Xencor, and Fate Therapeutics; speakers' bureau role for AstraZeneca, BMS/Celgene, Kite (a Gilead company), and TG Therapeutics; and research funding from AstraZeneca (institutional [Inst]), Xencor (Inst), Pharmacyclics (Inst), Curis (Inst), BMS (Inst), Celgene (Inst), MEI Pharma (Inst), Trillium Therapeutics (Inst), Kite/Gilead (Inst), Roche/Genentech (Inst), Fate Therapeutics (Inst), Takeda (Inst), Epizyme (Inst), Aptevo Therapeutics (Inst), Nurix (Inst), Loxo/Lilly (Inst), and Pfizer (Inst). J.M.V. reports consultancy for AbbVie and MEI Pharma; and research funding from Eli Lilly and Company, Epizyme, Kite, Loxo, and Novartis. N.N.S. reports participation on advisory boards and/or consultancy for Gilead-Kite, Bristol Myers Squibb (BMS)-Juno, Miltenyi Biomedicine, Lilly Oncology, Incyte, AbbVie, Cargo, BeiGene, Kite, Allogene, AstraZeneca, BMS, and Galapagos; research funding, travel support, and honoraria from Lilly Oncology, Genentech, and Miltenyi Biomedicine; and scientific advisory board participation for Tundra Therapeutics. P.L.Z. reports consulting or advisory role for MSD, Eusapharma, and Novartis; speakers’ bureau role for Celltrion, Gilead, Janssen-Cilag, BMS, Servier, MSD, AstraZeneca, Takeda, Roche, Eusapharma, Kyowa Kirin, Novartis, Incyte, and BeiGene; and is a member on an entity’s board of directors or advisory committees for Secura Bio, Celltrion, Gilead, Janssen-Cilag, BMS, Servier, Sandoz, MSD, AstraZeneca, Takeda, Roche, Eusapharma, Kyowa Kirin, Novartis, ADC Therapeutics, Incyte, and BeiGene. S.D.N. reports honoraria from ADC Therapeutics and Accrotech; served as a member on an entity’s board of directors or advisory committees for Merck-Data Safety Monitoring Committee; and reports research funding from Ono Pharmaceutical, Millennium Takeda, Loxo/Lilly, Genentech/Roche, Raphael, and Pharmacyclics. J.R.B. reports consultancy for AbbVie, Acerta/AstraZeneca, Alloplex Biotherapeutics, BeOne, Bristol Myers Squibb, EcoR1, Galapagos NV, Genentech/Roche, Grifols Worldwide Operations, InnoCare Pharma Inc, Loxo/Lilly, Magnet Biomedicine, Merck, and Pharmacyclics; research funding from BeOne, Gilead, iOnctura, Loxo/Lilly, MEI Pharma, Nagoon Therapeutics, and TG Therapeutics; and advisory board on the data safety monitoring board for Grifols Therapeutics. B.F. reports consultancy for AbbVie, ADC Therapeutics, AstraZeneca, BeiGene, BMS/Juno, Genentech, Genmab/AbbVie, Loxo Oncology, and Pharmacyclics; reports funding (research or clinical trial or other support) from AbbVie, Genentech, Genmab/AbbVie, and Loxo Oncology; and served as a member on an entity’s board of directors, speaker’s bureau, or its advisory committees for AbbVie, ADC Therapeutics, AstraZeneca, BeiGene, BMS/Juno, Genentech, Genmab/AbbVie, Loxo Oncology, and Pharmacyclics. C.C.C. reports having received honoraria/served as a consultant for AbbVie, Allogene, AstraZeneca, BeiGene, Genentech, Janssen, Lilly, MEI Pharma, MingSight, Octapharma, and TG Therapeutics; served on speaker’s bureaus for AbbVie, AstraZeneca, BeiGene, Genentech, Lilly; has stock in bluebird bio, Geron, and Pfizer; and has received research funding (Inst) from AbbVie, CarnaBio, and Lilly. S.M. reports honoraria from National Comprehensive Cancer Network (NCCN), Clinical Care Options, Curio Science, OncLive/MJH Life Sciences, and Research to Practice; consulting or advisory role for AbbVie, AstraZeneca, BeiGene, BMS, Genentech, and Janssen Pharmaceuticals; speakers bureau role with AstraZeneca, Eli Lilly–Loxo Oncology, and BeiGene; and research funding from AbbVie, AstraZeneca, BeiGene; Carna Biosciences, Juno-BMS, Janssen Pharmaceuticals, and Eli Lilly–Loxo Oncology. M.R.P. reports consulting fees from Kura, Accutar, and Mitsubishi; participating on a data safety monitoring board or advisory board with Lema, Nurix, Daiichi, Kura, and Janssen; leadership with ION Pharma; honoraria from Janssen Oncology; research funding from Loxo (Inst); and participating in a consulting or advisory role with Olema Pharmaceuticals, Daiichi Sankyo–UCB Japan, Kura Oncology, Accutar Biotech, and Kura. J.M.R. reports consultancy fees from Pharmacyclics, Jannsen, Genentech, GenMab, AstraZeneca, Morphosys, ADC Therapeutics, Epizyme, BeiGene, and AbbVie; research funding from Pharmacyclics, Velosbio, Loxo Oncology, Acerta, Oncternal Pharmaceuticals, Epizyme, and AbbVie; and honoraria from SeaGen. C.U. reports research funding from Abbvie, Pharmacyclics, Lilly, Gilead, Astrazenca, and consultancy from Abbvie, Genmab, ADC therapeutics, Beigene, Atara, and Astrazeneca. M.S.H. reports consultancy fees and honoraria from Pharmacyclics. B.T. reports receiving honoraria from Kite/Gilead and AbbVie; and travel and accommodation expenses from Kite/Gilead and AbbVie. A.D.Z. receives honoraria from NCCN, Curio Science, OncLive/MJH Life Sciences; reports consulting or advisory role for Genentech/Roche, Celgene, AstraZeneca, Dava Oncology, BeiGene, MEI Pharma, Kite (a Gilead company), Juno/Celgene/BMS, Sandoz, and Ono Pharmaceutical; receives research funding from Genentech/Roche, MEI Pharma, BeiGene, and AbbVie (Inst); and reports travel and accommodation expenses from Kite (a Gilead company), NCCN, and BeiGene. A.A. reports research funding from Incyte, BeiGene, and LOXO/Lilly; honoraria from Dr. Reddy; and advisory board membership for ADC Therapeutics, BeiGene, AbbVie, Lilly, Genentech, Amgen, Incyte, and Janssen. J.B.C. reports funding (research or clinical trial or other support) from Loxo Oncology, Takeda, Novartis, BMS/Celgene, Genentech, AstraZeneca, BioInvent, and Lam Therapeutics; and consulting fees from Loxo Oncology, BeiGene, Janssen, AbbVie, AstraZeneca, ADCT, and Kite. K.J.M. reports consulting fees from AbbVie, ADC, AstraZeneca, BMS, Caribou, Eli Lilly, Genentech, Genmab, Incyte, Janssen, Kite, Merck, MorphoSys, and Pharmacyclics. T.M. reports research grant funding from Janssen, AbbVie, and Pharmacyclics; consulting for Alexion, AstraZeneca, BeiGene, Janssen, MorphoSys, Roche, Sunesis, Lilly, and Sobi; and honoraria/travel support from AbbVie, AstraZeneca, BeiGene, Gilead, Janssen, Novartis/GlaxoSmithKline, Pharmacyclics, Roche, Sobi, and Alexion. J.A.W. reports consultancy from Newave, Loxo Oncology, BeiGene, AstraZeneca, AbbVie, Janssen, and Pharmacyclics and research funding from Schrodinger, Morphosys, Karyopharm, Janssen, and Pharmacyclics. D.L. reports consultancy and honoraria from AstraZeneca, Johnson and Johnson, Beigene, Roche, AbbVie, and Lilly. L.S. reports consulting or advisory role for Abbvie, Astrazeneca, BeOne Medicines Ltd, Johnson& Johnson, Lilly, Merck; and honoraria/travel support from Astrazeneca, BeOne Medicines Ltd, Johnson& Johnson. T.A.E. reports consultancy and honoraria from Beigene, AstraZeneca, Roche, Gilead, Kite (Gilead), Takeda, Johnson and Johnson, Loxo Oncology, Incyte, Autolus, Nurix, and Galapagos. F.S., Y.B., M.B., D.E.T., and P.A. are employees of and equity holder in Eli Lilly. C.Y.C. reports consulting fees/advisory fees/honoraria from Roche, Janssen, Gilead, AstraZeneca, Lilly, BeiGene, Menarini, Dizal, AbbVie, Genmab, Sobi, and BMS; research funding from BMS, Roche, AbbVie, MSD, and Lilly; and travel support from Lilly and BeiGene. The remaining authors declare no competing financial interests.
