# Supplementary material for: Pirtobrutinib, a highly selective, noncovalent (reversible) BTKi in R/R marginal zone lymphoma: phase 1/2 BRUIN study
Source: Blood Adv. 2026 Jan 20;10(7):2441–51. doi: 10.1182/bloodadvances.2025017489 (PMC13083716; doi:10.1182/bloodadvances.2025017489)
Supplement: Supplemental Figures [file BLOODA_ADV-2025-017489-mmc1.pdf]

## Supplemental Figures

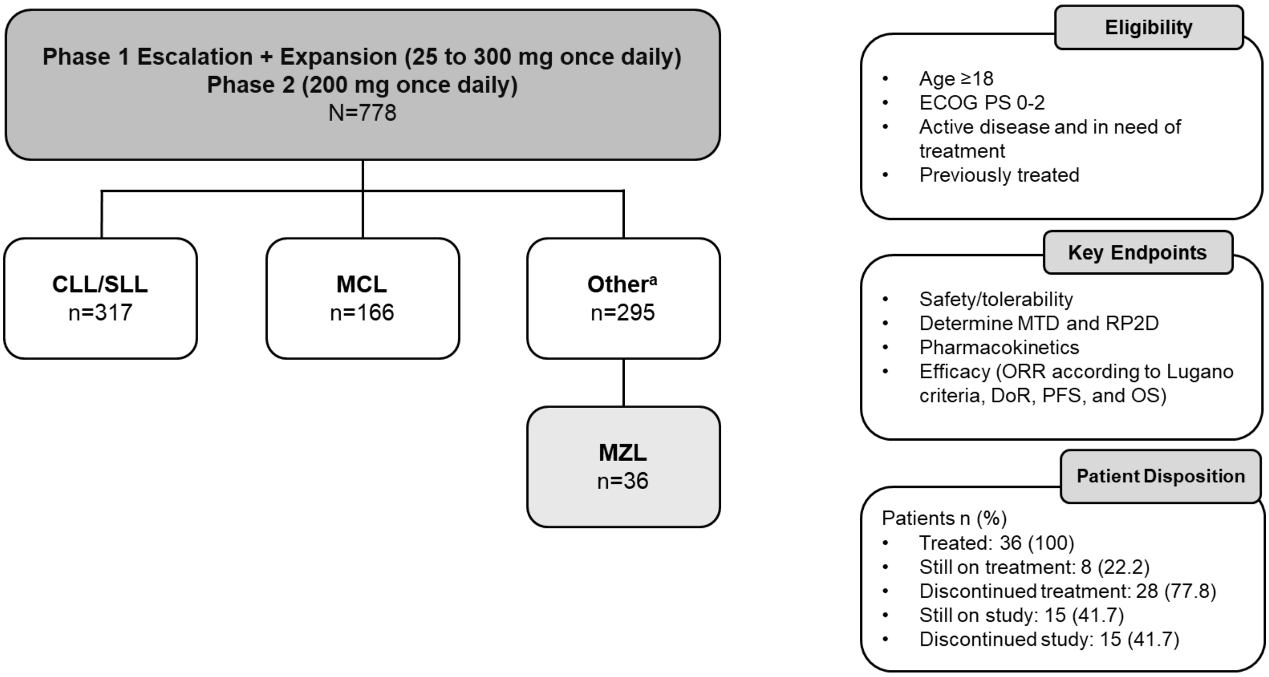


**Figure S1. Phase 1/2 BRUIN Study: Design, Eligibility, and Enrollment.**

Study schema of the BRUIN trial with key eligibility criteria and endpoints, a data cutoff of 27 Jan 2025.

[a]Other includes Richter transformation, marginal zone lymphoma, Waldenström macroglobulinemia, hairy cell leukemia, B-cell prolymphocytic leukemia, primary central nervous system lymphoma, and other transformations.

Abbreviations: CLL, chronic lymphocytic leukemia; DoR, duration of response; ECOG PS, Eastern Cooperative Oncology Group performance status; MCL, mantle cell lymphoma; MZL, marginal zone lymphoma, MTD, maximum tolerated dose; ORR, overall response rate; OS, overall survival; PFS, progression free survival; SLL, small lymphocytic lymphoma

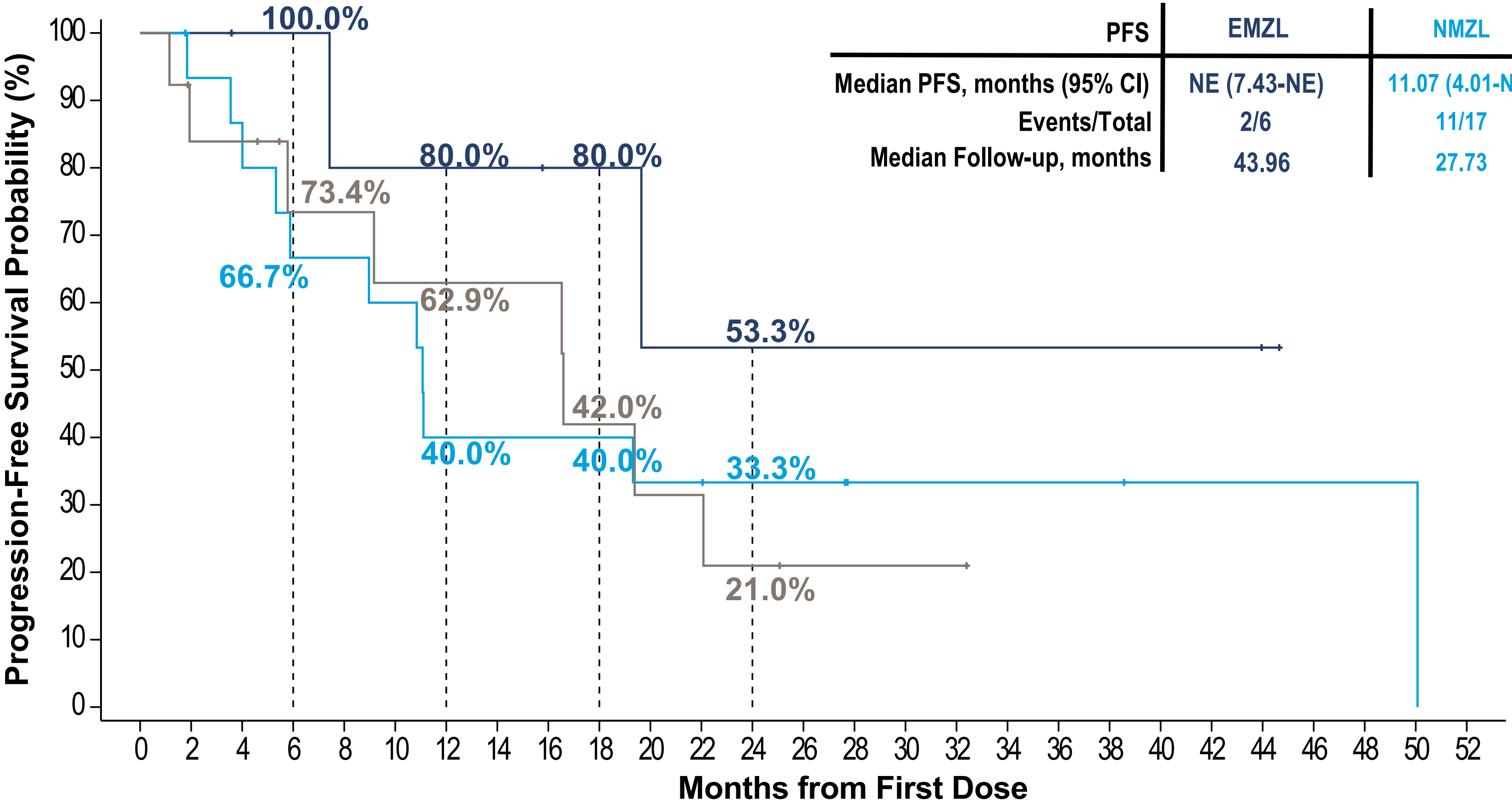


| PFS | EMZL | NMZL | SMZL |
|---|---|---|---|
| Median PFS, months (95% CI) | NE (7.43-NE) | 11.07 (4.01-NE) | 16.59 (1.94-22.08) |
| Events/Total | 2/6 | 11/17 | 8/13 |
| Median Follow-up, months | 43.96 | 27.73 | 25.07 |

| | | | | | | | | | | | | | | | | | | | | | | | | | | |
|---|---|---|---|---|---|---|---|---|---|---|---|---|---|---|---|---|---|---|---|---|---|---|---|---|---|---|
| EMZL: | 6 | 6 | 5 | 5 | 4 | 4 | 4 | 4 | 3 | 3 | 2 | 2 | 2 | 2 | 2 | 2 | 2 | 2 | 2 | 2 | 2 | 2 | 1 | 0 | 0 | 0 | 0 |
| NMZL: | 17 | 14 | 13 | 10 | 10 | 9 | 6 | 6 | 6 | 6 | 5 | 5 | 4 | 4 | 2 | 2 | 2 | 2 | 2 | 2 | 1 | 1 | 1 | 1 | 1 | 1 | 0 |
| SMZL: | 13 | 10 | 10 | 7 | 7 | 6 | 6 | 6 | 6 | 4 | 3 | 3 | 2 | 1 | 1 | 1 | 1 | 0 | 0 | 0 | 0 | 0 | 0 | 0 | 0 | 0 | 0 |

**Figure S2. PFS among patients with R/R MZL by MZL Subtype.**

Kaplan-Meier curve representing Investigator-assessed PFS in patients with R/R MZL by MZL subtype.

Abbreviations: CI, confidence interval; EMZL, extranodal marginal zone lymphoma; NE, not estimable; NMZL, nodal marginal zone lymphoma; SMZL, splenic marginal zone lymphoma.

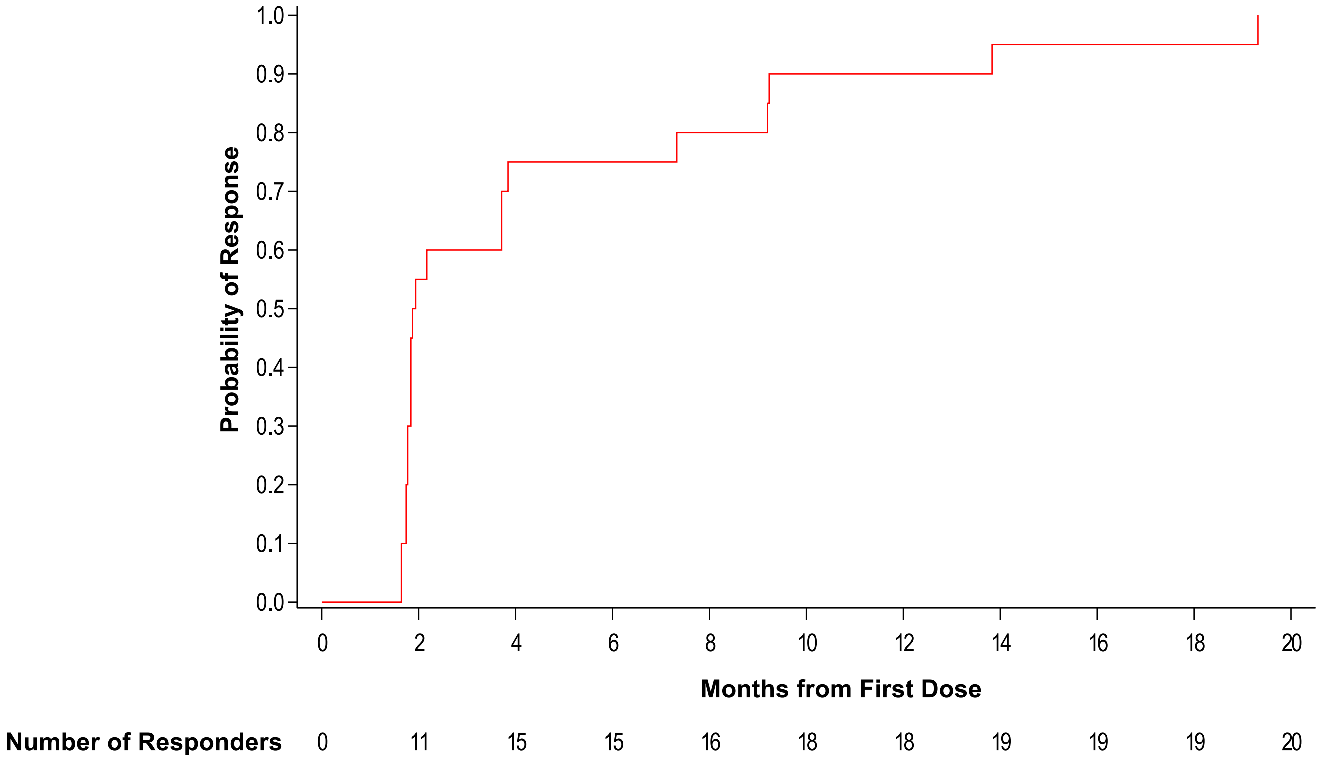


**Figure S3. Time to first response among patients with response**

Data cutoff of 27 Jan 2025.

## Reason for Discontinuation of any Prior BTKi

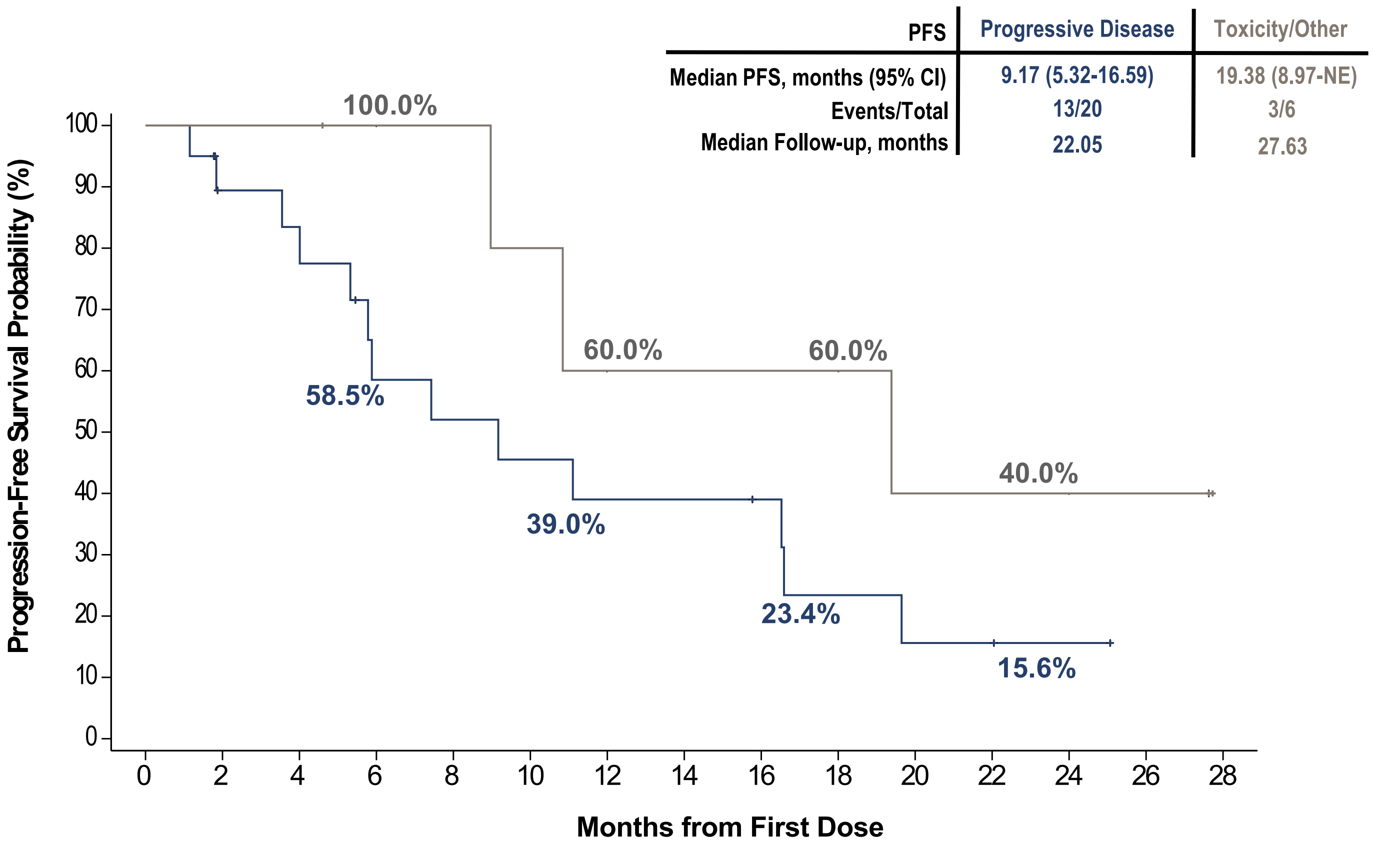


| | | | | | | | | | | | | | | |
|---|---|---|---|---|---|---|---|---|---|---|---|---|---|---|
| Progression Disease: | 20 | 15 | 14 | 9 | 8 | 7 | 6 | 6 | 5 | 3 | 2 | 2 | 1 | 0 | 0 |
| Toxicity/Other: | 6 | 6 | 6 | 5 | 5 | 4 | 3 | 3 | 3 | 3 | 2 | 2 | 2 | 2 | 0 |

**Figure S4. PFS stratified by reasons for discontinuation of prior BTKi therapy.**

Kaplan-Meier curve representing Investigator-assessed PFS in patients with R/R MZL according to reason for prior BTKi treatment discontinuation.

Abbreviations: BTKi, Bruton tyrosine kinase inhibitor; CI, confidence interval; NE, not estimable.

Data cutoff of 27 Jan 2025.
